# Association between glycemia and multi-vessel lesion in participants undergoing coronary angiography: a cross-sectional study

**DOI:** 10.3389/fcvm.2024.1435246

**Published:** 2024-07-17

**Authors:** Hezeng Dong, Zhaozheng Liu, Hao Chen, Jin Ba, Rui Shi, Qu Jin, Xiao Shao, Tenghui Tian, Jinzhu Yin, Liping Chang, Yue Deng

**Affiliations:** ^1^ College of Traditional Chinese Medicine, Changchun University of Traditional Chinese Medicine, Changchun, Jilin, China; ^2^ Cardiology Center, Affiliated Hospital of Changchun University of Traditional Chinese Medicine, Changchun, Jilin, China

**Keywords:** glycemia, multi-vessel lesion, coronary angiography, diabetes, Asian

## Abstract

**Background:**

This study aims to elucidate the association between glycemia and the occurrence of multi-vessel lesions in participants undergoing coronary angiography.

**Methods:**

We analyzed 2,533 patients with coronary artery disease who underwent coronary angiography. Of these, 1,973 patients, identified by the endpoint of multi-vessel lesions, were examined using univariate and multivariate logistic regression analyses to determine the relationship between glycemia levels and multi-vessel lesion occurrence.

**Results:**

The analysis included 1,973 participants, among whom 474 patients were identified with coronary multi-vessel lesions. Univariate logistic regression analysis demonstrated a positive correlation between glycemia and the occurrence of coronary multi-vessel lesions (OR 1.04; 95% CI 1.01–1.08; *p* = 0.02). The adjusted model indicated that for each unit increase in glycemia, the risk of developing coronary multi-vessel lesions increased by 4%, showing a significant correlation (*p* < 0.05). Subgroup analyses revealed that the impact of glycemia on multi-vessel lesions in patients with PCI varied according to gender, age, and smoking status, with the effect being more pronounced in men, older patients, and smokers.

**Conclusion:**

Our findings establish a significant association between glycemia and the incidence of multi-vessel lesions, particularly pronounced in male patients, individuals over 45, and smokers.

## Background

Advances in intravascular imaging and functional techniques, as well as coronary interventions (1), have led to a gradual increase in the detection rate of multi-vessel lesions in today's clinics. The European Society of Cardiology (ESC) has reported that more than 50% of patients with ST-segment elevation myocardial infarction (STEMI) have concomitant multibranch vasculopathy (2). Multi-vessel lesions often predict more serious adverse cardiovascular events (3). The risk of recurrent cardiovascular events is high even after interventional or pharmacological treatment (4). However, there are relatively few clinical studies on multi-vessel lesions, and there is a lack of effective predictive indicators for multi-vessel lesions (5), except for performing coronary angiography or intravascular ultrasound. We believe that it is crucial to identify and address the key factors in clinical practice. Timely intervention at an early stage is essential to prevent multi-vessel lesions and reduce the occurrence of acute coronary syndromes, lowering the risk of cardiovascular death. Individualized prevention and treatment protocols must be developed.

It is well established that diabetes mellitus and its complications represent a significant risk factor for coronary artery disease (6). Djupsjo, Kuhl, et al. demonstrated that patients with hyperglycemia exhibited a twofold increased risk of long-term cardiovascular death and a rate of cardiovascular events that were more than one times higher than that observed in patients with pre-diabetes (7). Jie Yang et al.'s study also found that glycosylated hemoglobin (HbA1c) and fasting blood glucose (FBG) are better at assessing the severity of coronary heart disease (CHD) in patients undergoing elective percutaneous coronary intervention (PCI) (8). Furthermore, Tütün U et al. demonstrated that uncontrolled glycemia levels not only increase perioperative complications but also the incidence of distal and middle coronary artery lesions. It is imperative to diagnose and aggressively control hyperglycemia before performing CABG (9). These studies confirm that glycemia aggravates the process of coronary atherosclerosis. However, direct clinical evidence of glycemia and multi-vessel lesions, a serious lesion in cardiovascular disease, is currently lacking, especially in Asia. This study is vital given the unique lifestyle and genetic characteristics of Asian populations. Our study will fill this gap by exploring the association between glycemia and multivessel disease in patients undergoing coronary angiography. The aim is to provide clinicians with more precise treatment options and to provide a scientific basis for cardiovascular risk management in diabetic patients.

## Method

**Figure 1:**
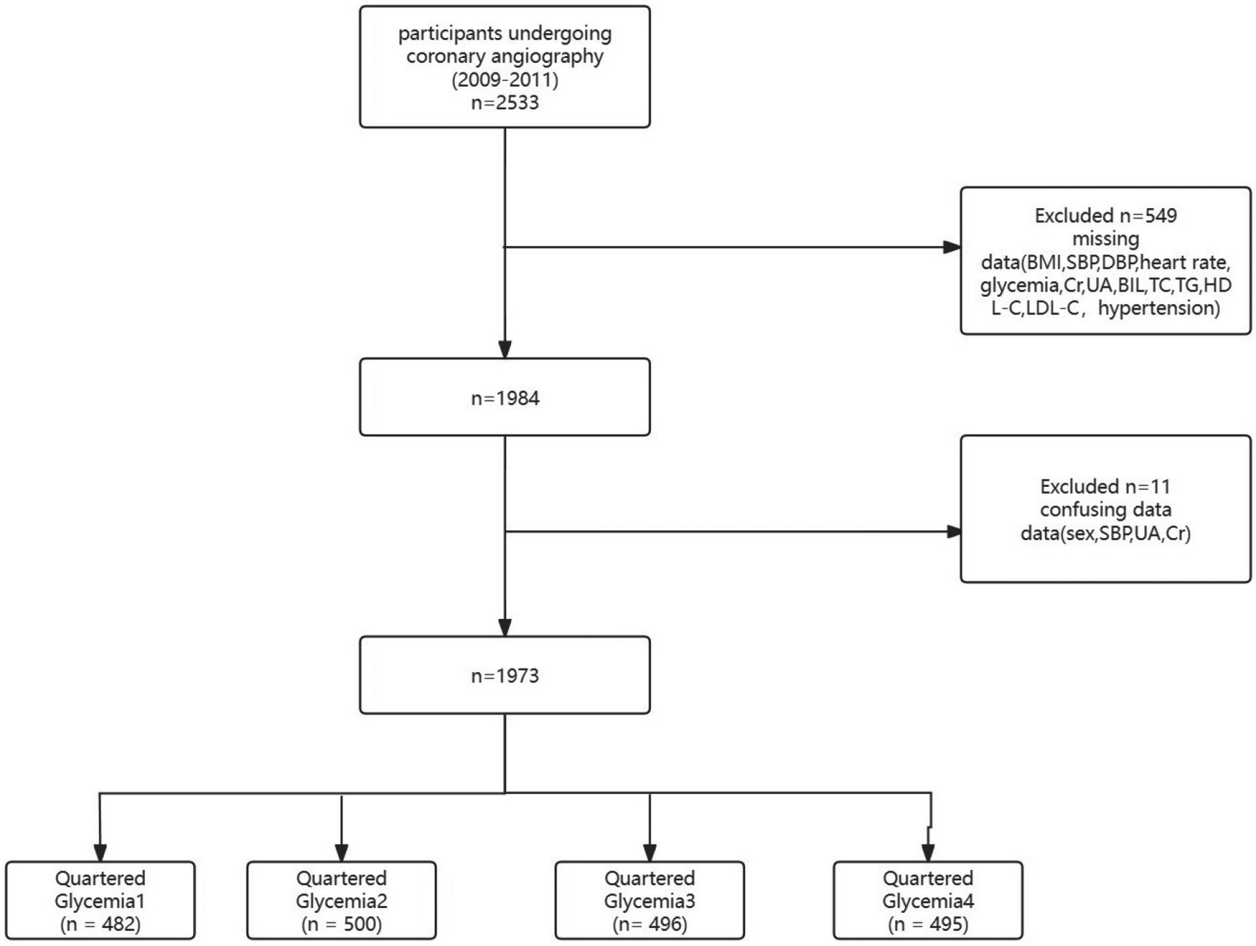
Flowchart of participant selection.

The participants in our study were all derived from patients who underwent coronary angiography between July 2009 and August 2011 at the First Affiliated Hospital of Zhengzhou University. Based on strict inclusion criteria, 1973 patients were included in this analysis after excluding incomplete and unclear data (Figure 1).

The primary endpoint of this study was a multi-vessel lesion, defined as the presence of ≥50% stenosis in at least two of the three major epicardial vessels. All participants underwent coronary angiography and quantitative analyses to characterize lesions according to standard methods. Furthermore, we collected comprehensive demographic and clinical data, which we then analyzed. All data was derived from a database containing demographic, clinical, angiographic, and procedural information. We also obtained data through patient visits, telephone interviews, and chart reviews, or by conducting clinical follow-ups. We then entered the data independently, and an independent committee adjudicated clinical events. The definitions of diabetes mellitus and hypertension as important risk factors for cardiovascular disease were based solely on clinical guidelines. Patients were defined as diabetic if they had a fasting blood glucose concentration of more than 6.1 mmol/L, a glycated hemoglobin level of more than 6.5%, or were receiving insulin or oral hypoglycaemic agents. Hypertension was defined as a systolic blood pressure of 140 mmHg or more and a diastolic blood pressure of 90 mmHg or more, or the current use of antihypertensive medications. A history of smoking was considered to be the presence of smoking within the previous ten years. Glycemia values were obtained from fasting blood samples at the time of admission, along with other laboratory tests including (Cr, UA, BIL, TC, TG, HDL-C, and LDL-C). All laboratory tests were collected and analyzed in compliance with the criteria (10).

The data that support the findings of this study are from Long-term follow-up results in patients undergoing percutaneous coronary intervention (PCI) with drug-eluting stents: results from a single high-volume PCI center [Dataset]. Dryad. https://doi.org/10.5061/dryad.13d31.

### Statistical analysis

In our study, we averaged participants’ glycemia levels into four quartiles: quartile 1 (*n* = 482), quartile 2 (*n* = 500), quartile 3 (*n* = 496) and quartile 4 (*n* = 495). We expressed categorical variables as numbers (*n*) and percentages (%) and assessed them using the chi-square test. Continuous variables are expressed as the mean ± standard deviation of normally distributed data. In addition, multiple imputation with multivariate imputation by chained equation was used for handling the missing values. We used univariate and multivariate regression analyses to examine the association between glycemia and multi-vessel lesions. In univariate analyses, we selected variables with a *p*-value <0.05, including age, gender, smoking, hypertension, DBP, HR, UA, and TG. We then adjusted for a variety of influences in multivariate analyses to validate the robustness of the results. Subgroup analyses were conducted using logistic models to determine the relationship between glycemia and multi-vessel lesions among subgroups, including gender, age, smoking status, and presence of diabetes. All analyses were performed using Free Statistics Approximation software version 1.9. A two-sided *P*-value of less than 0.05 was considered statistically significant.

## Result

### Study population and baseline characteristics

Our study involved 2,533 patients with coronary artery disease who underwent coronary angiography. After rigorous data screening, 1,973 participants were included in the final analysis. The cohort included 1,341 men and 632 women. The mean age was 59 years, and 474 participants were defined as having multi-vessel lesions. Glycemia was categorized into four quartiles, and a description of baseline characteristics revealed significant associations between glycemia and several key factors, including gender, age, BMI,hypertension, diabetes mellitus, and prevalence of multi-vessel lesions (Table 1).

**Table 1:** Baseline characteristics of the study participants.

	All participants (*n* = 1,973)	Quartile glycemia1 (*n* = 482)	Quartile glycemia2 (*n* = 500)	Quartile glycemia3 (*n* = 496)	Quartile glycemia4 (*n* = 495)	*p*	Statistic
Sex, *n* (%)						**0** **.** **004**	13.241
Female	632 (32.0)	135 (28)	144 (28.8)	168 (33.9)	185 (37.4)		
Male	1,341 (68.0)	347 (72)	356 (71.2)	328 (66.1)	310 (62.6)		
Age (years)	59.9 ± 11.1	58.7 ± 12.1	59.7 ± 11.0	60.6 ± 10.9	60.8 ± 10.2	**0**.**013**	3.592
Mean ± SD							
Hypertension *n* (%)						**0**.**001**	16.082
No	975 (49.4)	264 (54.8)	261 (52.2)	238 (48)	212 (42.8)		
Yes	998 (50.6)	218 (45.2)	239 (47.8)	258 (52)	283 (57.2)		
DM, *n* (%)						**<0**.**001**	492.204
No	1,553 (78.7)	448 (92.9)	466 (93.2)	422 (85.1)	217 (43.8)		
Yes	420 (21.3)	34 (7.1)	34 (6.8)	74 (14.9)	278 (56.2)		
Heart.failure, *n* (%)						0.723	1.324
No	1,744 (88.4)	427 (88.6)	445 (89)	442 (89.1)	430 (87)		
Yes	228 (11.6)	55 (11.4)	55 (11)	54 (10.9)	64 (13)		
Angina, *n* (%)						0.313	3.561
No	1,745 (88.4)	415 (86.1)	444 (88.8)	444 (89.5)	442 (89.3)		
Yes	228 (11.6)	67 (13.9)	56 (11.2)	52 (10.5)	53 (10.7)		
AMI, *n* (%)						0.307	3.604
No	1,880 (95.3)	465 (96.5)	476 (95.2)	474 (95.6)	465 (93.9)		
Yes	93 (4.7)	17 (3.5)	24 (4.8)	22 (4.4)	30 (6.1)		
Smoking, *n* (%)						0.06	7.398
No	1,322 (67.0)	312 (64.7)	319 (63.8)	340 (68.5)	351 (70.9)		
Yes	651 (33.0)	170 (35.3)	181 (36.2)	156 (31.5)	144 (29.1)		
SBP (mmHg)	104.5 ± 28.5	108.8 ± 28.1	102.5 ± 28.2	106.6 ± 29.0	100.1 ± 28.0	**<0**.**001**	9.372
Mean ± SD							
DBP (mmHg)	77.3 ± 11.9	78.0 ± 11.6	76.0 ± 11.6	77.4 ± 12.1	78.0 ± 12.2	**0**.**031**	2.964
Mean ± SD							
EF, Mean ± SD	61.0 ± 7.8	61.8 ± 7.4	60.8 ± 8.1	61.0 ± 7.3	60.4 ± 8.2	**0**.**024**	3.166
BMI (kg/m^2^), Mean ± SD	24.1 ± 3.6	24.0 ± 3.3	23.8 ± 3.6	24.4 ± 3.6	24.3 ± 3.8	**0**.**027**	3.068
Heart.rate,	72.1 ± 11.5	69.8 ± 10.8	71.1 ± 10.1	73.0 ± 11.6	74.4 ± 12.9	**<0**.**001**	15.778
Mean ± SD							
Cr (*μ*mol/L)	72.0 ± 30.2	73.3 ± 25.5	72.3 ± 20.5	73.0 ± 40.1	69.2 ± 31.1	0.133	1.867
Mean ± SD							
UA (μmol/L)	304.2 ± 92.5	306.4 ± 87.0	308.0 ± 84.3	310.4 ± 100.5	291.9 ± 96.3	**0**.**007**	4.034
Mean ± SD							
BIL (mg/dl)	9.8 ± 7.6	9.4 ± 4.6	9.5 ± 5.2	10.4 ± 12.3	10.0 ± 5.7	0.160	1.723
Mean ± SD							
TC (Mmol/L)	4.3 ± 1.1	4.1 ± 1.0	4.2 ± 1.0	4.3 ± 1.1	4.4 ± 1.1	**<0**.**001**	9.736
Mean ± SD							
TG (Mmol/L)	1.9 ± 1.4	1.6 ± 0.8	1.8 ± 1.2	2.1 ± 1.9	2.2 ± 1.4	**<0.001**	14.313
Mean ± SD							
HDL,C (Mmol/L)	1.1 ± 0.3	1.1 ± 0.3	1.1 ± 0.3	1.1 ± 0.3	1.0 ± 0.3	0.215	1.493
Mean ± SD							
LDL.C (Mmol/L)	2.7 ± 0.9	2.5 ± 0.9	2.7 ± 0.9	2.7 ± 0.9	2.8 ± 1.0	**<0**.**001**	6.921
Mean ± SD						** **	
Multi-vessel lesion *n* (%)						**0**.**003**	13.998
No	1,499 (76.0)	392 (81.3)	379 (75.8)	376 (75.8)	352 (71.1)	** **	
Yes	474 (24.0)	90 (18.7)	121 (24.2)	120 (24.2)	143 (28.9)	** **	

Data are shown as mean ± standard deviation (SD) or median (IQR) for continuous variables and proportions (%) for categorical variables. Sex, Age, Hypertension, DM, Heart failure, Angina, Acute myocardial infarction, Smoking, SBP, DBP, EF,BMI,Heart rate, Cr, UA, BIL, TC, HDL, C, LDL.C, Multi-vessel lesion *P*-values in bold are <0.05.

### Univariate and multifactorial analysis

In univariate analysis, age, hypertension, diabetes mellitus, glycemia level, uric acid level, and triglycerides were significantly associated with coronary multi-vessel lesion (Table 2).

**Table 2:** Univariate analysis for overall population.

Variable	OR_95CI	*P*_value
Sex = female, *n* (%)	0.94 (0.75∼1.17)	0.56
Age (years)	1.03 (1.02∼1.04)	**<0** **.** **001**
Hypertension, *n* (%)	1.31 (1.06∼1.61)	**0**.**011**
DM, *n* (%)	1.85 (1.46∼2.34)	**<0**.**001**
Smoking, *n* (%)	0.97 (0.78∼1.21)	0.788
SBP (mmHg)	1 (1∼1.01)	0.317
DBP (mmHg)	1.01 (1∼1.02)	**0**.**012**
Heart.rate (Bpm)	1.01 (1∼1.02)	0.13
Glycemia (Mmol/L)	1.04 (1.01∼1.08)	**0**.**02**
Cr (μmol/L)	1 (1∼1)	0.358
UA (μmol/L)	1 (1∼1)	**0**.**023**
BIL (mg/dl)	1.01 (0.99∼1.02)	0.394
TC (Mmol/L)	1.02 (0.93∼1.12)	0.693
TG (Mmol/L)	1.09 (1.01∼1.17)	**0**.**018**
HDL.C (Mmol/L)	1.06 (0.76∼1.46)	0.746
LDL.C (Mmol/L)	1.02 (0.91∼1.14)	0.713

OR, odds ratio; CI, confidence interval; SD, standard deviation. Abbreviations as in Table 1. *P* values in bold are <0.05.

To further elucidate the relationship between participants' glycemia and multi-vessel lesions, we performed a multifactorial logistic analysis. In the unadjusted model, there was a significant correlation between glycemia and coronary multivessel disease, with a 4% increase in the risk of multi-vessel lesions for each unit increase in glycemia (OR: 1.04, *P* = 0.02). This relationship remained significant after adjusting for sex, age, smoking, hypertension, diastolic blood pressure, heart rate, uric acid, and triglycerides. (Adj. OR: 1.04, *P* = 0.039) (Table 3).

**Table 3:** Multivariate analysis for overall population.

Variable	Model 1	Model 2	Model 3	Model 4
*n* total	1,973	1,973	1,973	1,973
*n* event_%	474 (24)	474 (24)	474 (24)	474 (24)
crude OR (95%CI)	1.04 (1.01∼1.08)	1.04 (1.01∼1.08)	1.04 (1.01∼1.08)	1.04 (1.01∼1.08)
crude *P*_value	**0.02**	0.02	0.02	0.02
adj. OR (95%CI)		1.04 (1∼1.07)	1.04 (1∼1.07)	1.04 (1∼1.07)
adj. *P*_value		**0.024**	**0.025**	**0.039**

Model 1: no adjusted.

Model 2: Adj: Model 1 + Sex + age.

Model 3: Adj: Model 2 + smoking + hypertension.

Model 4: Adj: Model 3 + DBP + HR + UA + TG.

*P* values in bold are <0.05.

**Figure 2:**
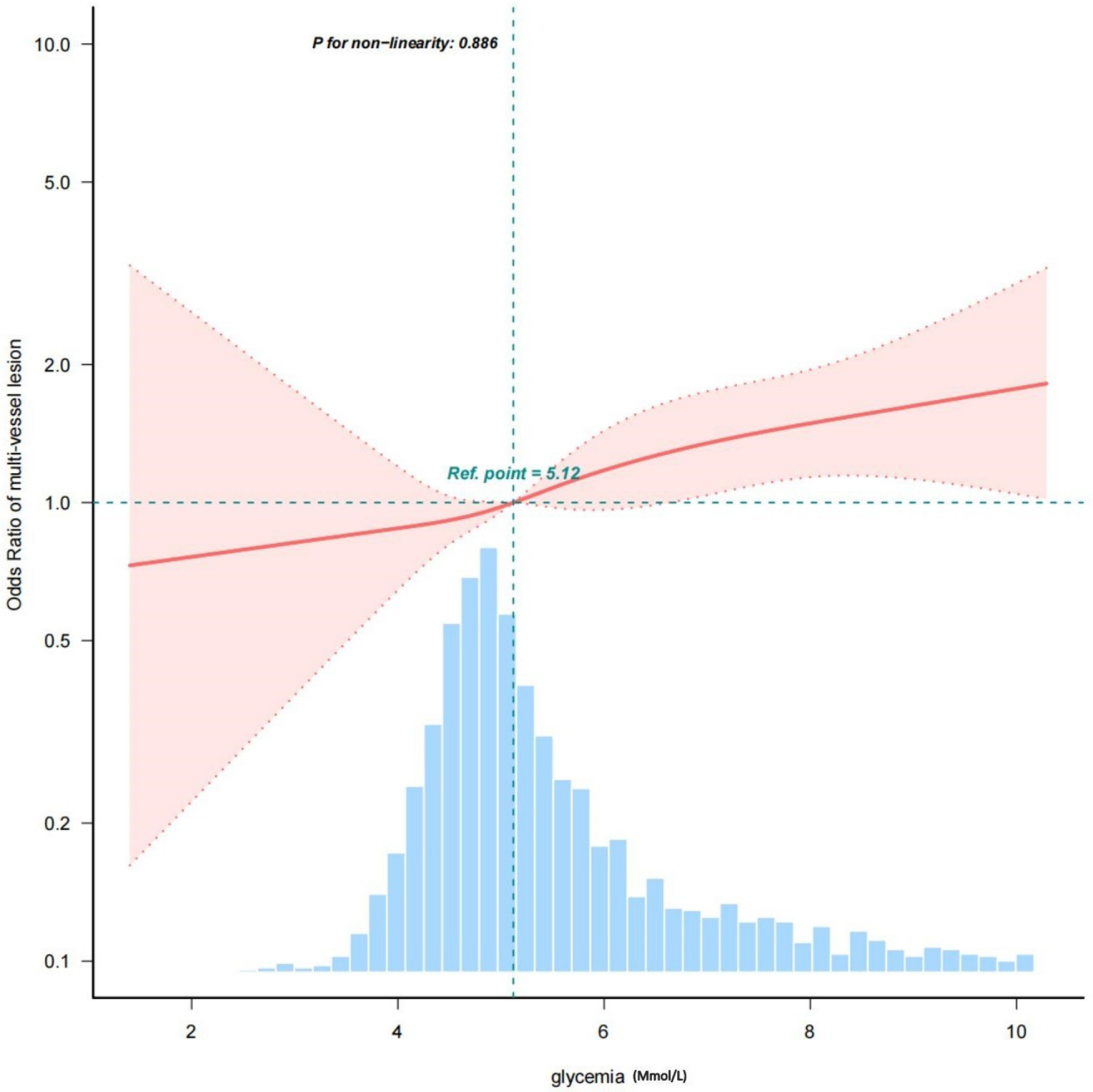
A linear relationship between glycemia and multi-vessel mesion.

These results are clear: glycemia is an important risk factor for the development of coronary multi-vessel lesion. After adjusting for various covariates, we observed a linear relationship between glycemia and multi-vessel lesions, with the risk of developing multi-vessel lesions progressively increasing with increasing glycemia levels (Figure 2).

### Subgroup analysis

**Figure 3:**
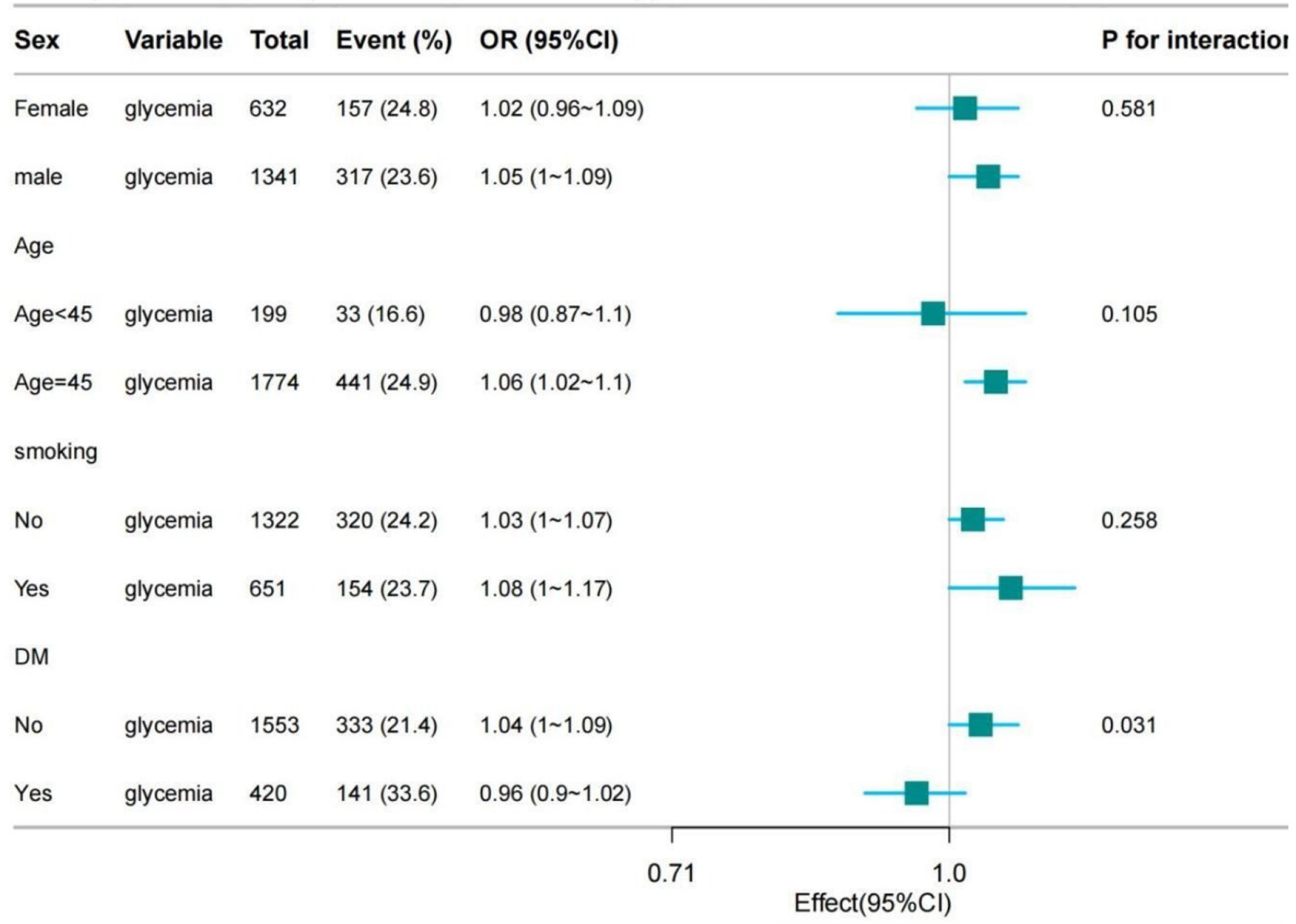
Strtification analysis on the association between glycemia and multi-vessel lesion.

To clarify the relationship between glycemia and multi-vessel lesion in different age, gender and smoking status, we conducted further subgroup analyses. These showed that glycemia and multi-vessel lesion had a more significant association in males (*p* = 0.031) compared to females. The analyses showed a significant association between glycemia and multi-vessel lesion in those aged ≥ 45 years (*p* = 0.008). Furthermore, smokers showed a stronger correlation (*p* = 0.038) compared to non-smokers (*p* = 0.085). Due to the lack of information on medications taken by patients prior to admission, diabetic patients who were regularly taking hypoglycaemic medications prior to admission would have resulted in relatively low fasting glycemia values on admission, which would have had an impact on our findings. Consequently, we grouped the patients by previous diabetes or not, and found that compared to diabetic patients, blood glucose and multi-vessel lesion were yet more significantly associated among non-diabetic patients. This shows that even non-diabetics should be aware of glycemia changes. The association between glycemia and multi-vessel lesion was stronger in non-diabetics among the participants who underwent coronary angiography. Therefore, close monitoring of glycemia is essential to prevent adverse cardiovascular events, regardless of previous diagnosis of diabetes mellitus. In conclusion, the findings demonstrate the complexity of cardiovascular risk factors and their differential impact in different patient subgroups. This stratified analysis will help to develop a more personalised management strategy for patients (Figure 3 and Table 4).

**Table 4:** Subgroup analysis for association between glycemia and multi-vessel lesion.

Subgroup	*n* total	*n* event_%	crude OR_95CI	crude *P*_value	P for interaction_1	P for interaction_2
Sex						
Female	632.0	157 (24.8)	1.02 (0.96∼1.09)	0.477	0.581	0.585
Male	1,341.0	317 (23.6)	1.05 (1∼1.09)	0.031		
Age (years)						
Age < 45	199.0	33 (16.6)	0.98 (0.87∼1.1)	0.688	0.105	0.208
Age ≥ 45	1,774.0	441 (24.9)	1.06 (1.02∼1.1)	0.008		
Smoking						
No	1,322.0	320 (24.2)	1.03 (1∼1.07)	0.085	0.258	0.253
Yes	651.0	154 (23.7)	1.08 (1∼1.17)	0.038		
DM						
No	1,553.0	333 (21.4)	1.04 (1∼1.09)	0.074	0.031	0.038
Yes	420.0	141 (33.6)	0.96 (0.9∼1.02)	0.191		

OR, odds ratio; CI, confidence interval; SD, standard deviation; Other abbreviations as in Table 1.

### Epidemiology and significance of multivessel lesions

The incidence of multi-vessel lesions is increasing in clinical practice and is a matter of considerable concern in current clinical cardiovascular disease research. There is a clear association between multi-vessel lesions and a wide range of adverse cardiovascular outcomes (11). Dziewierz, Siudak et al. reported that multi-vessel lesions were present in approximately 40%–65% of patients with ST-segment elevation myocardial infarction (STEMI) or complete coronary occlusion, as well as other coronary artery disease (12). A prospective randomised, multicentre, open-label and controlled clinical trial enrolled 396 patients and found that 52% had multivessel disease (13). Furthermore, Tindale A et al. demonstrated that patients with multi-vessel lesion treated with CR who developed STEMI with cardiogenic shock (defined as lactic acid ≥2 mmol/L) had a higher mortality rate (14). This finding is in line with Sorajja, Bernard J. et al., who observed that three-vessel disease significantly predicted cardiovascular mortality and risk of reinfarction (15). These findings demonstrate that multi-vessel lesion is a serious and widespread cardiovascular disease process, that the number of patients who develop multi-vessel lesions is enormous, and that understanding and managing multi-vessel lesions to avoid adverse cardiovascular events is of the utmost importance.

Glycaemia is clearly associated with several cardiovascular diseases (16). Our study definitively confirms the link between elevated glycaemia and cardiovascular disease. This observation is in line with the findings of Xiang Wang et al. who concluded that the TyG index can be a valuable predictor of CAD severity, especially for patients with prediabetes (17). Furthermore, a study by Iijima R, et al. demonstrated that Patients with diabetes often accelerate atherosclerotic thrombosis, resulting in early, widespread, and rapidly progressing coronary artery disease (18). Tong Zhao et al. concluded that hyperglycaemia was an independent predictor of severe coronary artery disease in non-diabetic patients (19). Our study definitively confirms that the association of glycemia with multi-vessel lesions is more significant in non-diabetic patients. Clinicians must be aware of this and provide appropriate early intervention to prevent adverse cardiovascular events.

### Unique considerations for Asian populations

It is crucial to note that our study differs from previous studies in two key ways. Firstly, we have a larger sample size. Secondly, we only include Asian populations. This is because Asia is an important region for the development of cardiovascular disease and diabetes worldwide. This may be due to unique lifestyle and genetic influences, among other factors. Expert discussions at the WHO have made it clear that, at a BMI below the existing WHO overweight threshold (≥25 kg/m^2^), Asians are at a much higher risk of developing type 2 diabetes and cardiovascular disease (20). It is therefore of great importance to conduct a study of diabetes and cardiovascular disease in Asia. By identifying the link between glycemia and multivessel disease, physicians will be able to more accurately assess a patient's risk of developing multivessel disease.

### Limitations and outlook

Our study is comprehensive, but it has limitations. Our study was a cross-sectional investigation, so even after rigorous data screening, potential confounders could not be completely eliminated. This may limit the generalisability of the findings, but we included a relatively large number of participants, and the results are still instructive for future studies to provide a basis for a deeper understanding of the relationship between diabetes mellitus and cardiovascular disease. Longitudinal studies are needed to understand the long-term effects of glycemia on cardiovascular occurrence and prognosis in patients with multivessel disease. It is also crucial to include participants from more regions and ethnicities to raise awareness of glycaemic control in all regions. Clinicians must be vigilant about the glycaemic status of their patients, as this is a key factor in the assessment and management of cardiovascular risk.

## Conclusion

Our study definitively demonstrated a linear relationship between glycemia and multivessel disease in patients undergoing coronary angiography. Even after adjusting for study-related confounders, the results remained significant. This indicates that the risk of multi-vessel lesion increases progressively with increasing glycemia levels. Our study provides unquestionable evidence that glycemia control is crucial for the prevention and treatment of multi-vessel lesions. It also offers invaluable insights for improving risk assessment and management of cardiovascular disease. These findings have significant implications for public health policy development and optimisation of clinical care, particularly in areas with a high prevalence of diabetes and cardiovascular disease.

## Data Availability

The datasets presented in this study can be found in online repositories. The names of the repository/repositories and accession number(s) can be found below: https://doi.org/10.5061/dryad.13d31.
